# A Low-Cost Inkjet-Printed Aptamer-Based Electrochemical Biosensor for the Selective Detection of Lysozyme

**DOI:** 10.3390/bios8010007

**Published:** 2018-01-15

**Authors:** Niazul Islam Khan, Alec G. Maddaus, Edward Song

**Affiliations:** 1 Department of Electrical and Computer Engineering, University of New Hampshire, Durham, NH 03824, USA; mk1125@wildcats.unh.edu; 2 Department of Chemical Engineering, University of New Hampshire, Durham, NH 03824, USA; agm11@wildcats.unh.edu; 3 Center for Advanced Materials and Manufacturing Innovation, University of New Hampshire, Durham, NH 03824, USA

**Keywords:** biosensor, aptamer, lysozyme, electrochemical impedance spectroscopy, inkjet printing, point-of-care

## Abstract

Recently, inkjet-printing has gained increased popularity in applications such as flexible electronics and disposable sensors, as well as in wearable sensors because of its multifarious advantages. This work presents a novel, low-cost immobilization technique using inkjet-printing for the development of an aptamer-based biosensor for the detection of lysozyme, an important biomarker in various disease diagnosis. The strong affinity between the carbon nanotube (CNT) and the single-stranded DNA is exploited to immobilize the aptamers onto the working electrode by printing the ink containing the dispersion of CNT-aptamer complex. The inkjet-printing method enables aptamer density control, as well as high resolution patternability. Our developed sensor shows a detection limit of 90 ng/mL with high target selectivity against other proteins. The sensor also demonstrates a shelf-life for a reasonable period. This technology has potential for applications in developing low-cost point-of-care diagnostic testing kits for home healthcare.

## 1. Introduction

Aptamers hold great interest to the scientific community due to their versatile advantages with regards to biosensing. Their many advantages, including high affinity and binding efficiency to the target analyte, chemical and thermal stability, resistance to harsh environmental conditions, long shelf-life, mass producibility at low-cost, and reusability make aptamers attractive alternatives to their natural counterparts, such as antibodies and enzymes [1,2]. Selected in vitro by a well-established technique known as the Systematic Evolution of Ligands by EXponential enrichment (SELEX), aptamers can be used for the selective detection of a broad range of analytes including proteins, peptides, amino acids, drugs, metal ions, and even whole cells [3]. The detection of lysozyme has received much attention among researchers because of its various significances in medicine, as well as in the food industry. Having a molecular weight of 14.4 kDa with a primary sequence containing 129 amino acids and an isoelectric point of 11.0, lysozyme is a ubiquitous enzyme widely available in diverse organisms such as bacteria, bacteriophages, fungi, plants and mammals [4,5]. Lysozyme also plays an important role as a biomarker for diagnosing diseases such as breast cancer [6], Alzheimer’s disease [7], rheumatoid arthritis [8], malaria [9], AIDS [10], tuberculosis and leprosy [11], sarcoidosis [12], and Crohn’s disease [13]. Typically, the concentration of lysozyme in a healthy person’s saliva is 13.8 µg/mL [14], whereas the concentration is 0.463–2.958 µg/mL in a healthy person’s serum [1].

Existing aptamer-based biosensors use different detection schemes such as high-performance liquid chromatography (HPLC), quartz crystal microbalance (QCM), surface plasmon resonance (SPR), and fluorescence-based optical detection. However, these methods suffer from several drawbacks as they are often time-consuming, expensive, operated by highly-trained technicians, and performed in a laboratory setting [2,15]. However, as an alternative, electrochemical detection offers the potential for a rapid, low-cost, and sensitive detection of the target species. Especially, electrochemical impedance spectroscopy (EIS) has proven to be a powerful and sensitive tool for investigating the features of surface-modified electrodes [15]. EIS can be used to monitor the changes in the electrical properties of the biosensor at different stages, including different fabrication steps as well as the detection of target recognition events [16]. The unique advantages of EIS include the ease of signal quantification, the ability to separate the surface binding events from the solution impedance, non-invasive measurement, real-time monitoring and label-free detection, making it an effective tool for electrochemical interrogation [16].

An important step towards the fabrication of the aptamer-based electrochemical biosensor is the immobilization of the aptamer probes onto the working electrode so that the target recognition by the aptamer can be transduced into a measurable electrical signal. Rohrbach et al. [2] developed a lysozyme biosensor where the covalent coupling between the carboxylic groups of CNT and the amino groups linked to the aptamer was used to immobilize the aptamer. An EDC/NHS coupling-based immobilization technique has been exploited by Kara et al. to develop an aptamer-based biosensor for the detection of thrombin with a detection limit of 105 pM using EIS [17]. Others have used thiol-gold binding [14,15,18], biotin-avidin affinity-based binding [19], and surface adsorption [20] to immobilize aptamers on the respective electrodes. However, such approaches can be difficult to reproduce, often require complex chemistry, lack control over aptamer density, and may not be suitable for large-scale manufacturing and mass production.

Herein, we explore the possibility of using the inkjet-printing technique for a reliable and reproducible aptamer immobilization method. We propose the use of a dispersed CNT-aptamer complex as a printable ink to be deposited on the electrode. The ink exploits the strong π–π stacking interaction between the nucleotide bases of the single stranded DNA and the sidewalls of the CNT [21]. Inkjet-printing is finding applications in areas such as flexible electronics, disposable sensors, and wearable devices [22]. Particularly, due to its on-demand printability of the devices, inkjet-printed sensors can potentially be used as point-of-care (POC) diagnostic tools and disposable testing kits. In contrast to other existing aptamer immobilization techniques, the proposed approach of inkjet-printing offers many advantages, including mass producibility, uniform deposition of materials, fully automated process, and high throughput [22,23,24]. We also demonstrate in this work that the aptamer density can be controlled by utilizing the number of printing layers. After the deposition of the CNT-aptamer ink, the sensor is then used for the detection of lysozyme using EIS. The binding affinity of our aptamer probe to lysozyme was confirmed by the square wave voltammetric techniques using methylene blue (MB)-labeled aptamers (see Appendix B).

**Figure 1:**
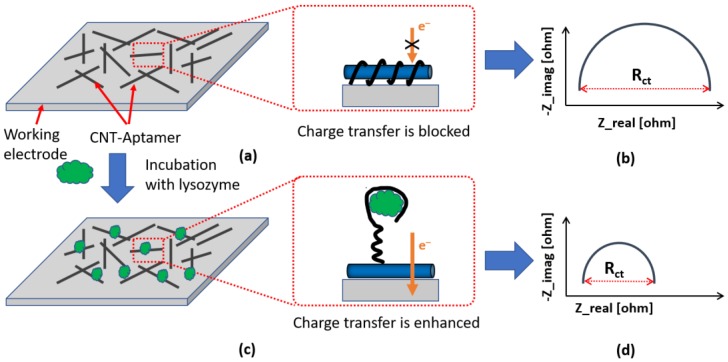
Working principle of the aptamer-based biosensor. Initially, the printed sensor blocks the charge transfer from the redox probe to the electrode due to the negative backbone of DNA bases (**a**); and the corresponding Nyquist curve (**b**). When exposed to the lysozyme, the anti-lysozyme aptamer unwraps itself from the carbon nanotube (CNT) and binds to the lysozyme, opening up the current path for enhanced charge transfer (**c**); and the corresponding Nyquist curve (**d**).

Figure 1 presents the working principle of our proposed biosensor. Figure 1a shows the CNT-aptamer complex deposited on the working electrode. Due to the negatively charged backbone as well as the insulating property of the aptamers, the charge (electron) transfer from the redox probe (e.g., ferro- and ferri-cyanide) to the electrode is hindered, i.e., the charge transfer resistance (R_ct_) is large as illustrated by the larger diameter of the Nyquist plot in Figure 1b. When the sensor is exposed to lysozyme as shown in Figure 1c, the aptamer unwraps itself from the CNT due to its preferential binding to the lysozyme. This conformational change in the aptamers opens up the path for electrons to easily flow from the redox probes to the working electrode, resulting in an enhancement in the rate of charge transfer and thus a reduction in R_ct_, as shown in Figure 1d with a smaller radius of the Nyquist curve.

## 2. Materials and Methods

### 2.1. Materials

Multi-walled carbon nanotubes (>99.9% purity, 30–50 nm outer diameter, 10–20 µm length) modified with carboxyl functional groups (–COOH) were purchased from Cheap Tubes (Cambridgeport, VT, USA) and used without further modification. Single-stranded anti-lysozyme DNA oligonucleotides (sequence designed by Ellington and co-workers [25]) were synthesized by Sigma-Aldrich (St. Louis, MA, USA). The sequence of the oligonucleotides is: 5′-ATC AGG GCT AAA GAG TGC AGA GTT ACT TAG-3′. Methylene blue (MB)-labeled and thiolated DNAs with the same sequence (thiol group attached at the 5′ end MB attached at 3′ end) were purchased from LGC Biosearch Technologies (Novato, CA, USA). Lysozyme from chicken egg white, bovine serum albumin, and thrombin were also purchased as lyophilites from Sigma-Aldrich. The stock solutions were prepared by dissolving the lyophilites in fresh ultrapure triple-distilled water and stored at −20 °C until used. The diluted solutions of proteins were prepared in 50 mM phosphate buffer solution (PBS, pH 7.4, Sigma-Aldrich).

### 2.2. Electrochemical Assay

The Bio-Logic VSP-300 potentiostat was used for the electrochemical measurements. All experiments were performed using screen-printed carbon electrodes (SPCEs) purchased from DropSens (Spain). These disposable SPCEs consist of three electrodes: a carbon working electrode (WE), a carbon counter electrode (CE) and a silver pseudo reference electrode (RE). The WE is circular in geometry with a diameter of 4 mm.

### 2.3. Ink Preparation

First, 0.25 mg/mL of multi-walled carbon nanotubes (MWCNTs) were mixed with 5 µM lysozyme binding aptamer in 30% N-methyl pyrrolidone (NMP) solution. Next, the mixture was sonicated using an ultrasonic bath sonicator for 2 h and then centrifuged at 6000 rpm for 30 min in order to remove any MWCNT aggregates. Afterwards, the supernatant was collected and loaded into the ink cartridge for printing. The unused ink was stored in a refrigerator at 4 °C.

### 2.4. Inkjet-Printing

The Fujifilm Dimatix Materials Printer (DMP-2831) was used for the inkjet-printing. It uses a 16-jet Dimatix Materials Cartridge with 10 pL drop volumes. The minimum patterning resolution of this printer was reported to be 20 µm [26]. Each device was printed with 5 layers of the CNT-aptamer ink. The ink was printed at a voltage of 40 V, a nozzle temperature of 35 °C and a 5 kHz jetting frequency. The amount of ink printed per layer is estimated to be approximately 315 nL (see Appendix A for detailed calculation).

### 2.5. Removal of the Unbound Aptamers

**Figure 2:**
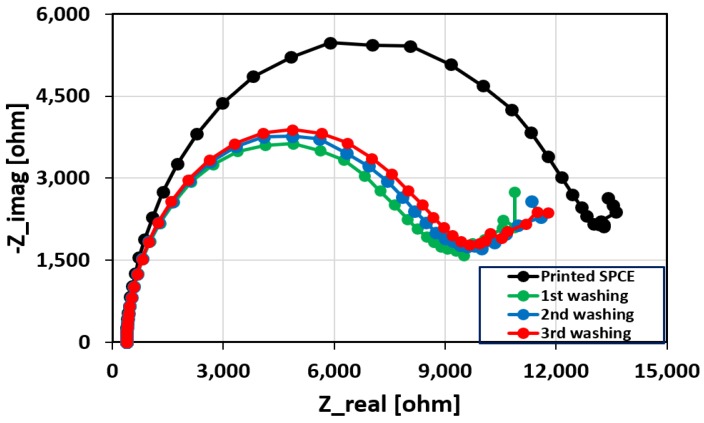
The effect of washing on the printed sensor. After the first wash, the R_ct_ value obtained from the Nyquist curve drops by approximately 31%. Subsequent washes do not significantly change the radius of the Nyquist curves, suggesting that the remaining aptamers are securely attached to the CNTs.

After the printing process, the SPCE devices were dried on a hotplate at 35 °C and gently washed with deionized (DI) water to remove any unbound DNAs. The effect of washing is presented in Figure 2.

It can be seen that the radius of the Nyquist curve corresponding to the first wash drops significantly and remains stable for the subsequent washes. This indicates that the majority of the unbound or loosely bound aptamers have been removed after the first rinsing procedure during which 31% reduction in the charge transfer resistance has been observed.

### 2.6. EIS Measurements

The electrochemical impedance spectroscopy (EIS) measurements were performed with 1 mM K_4_[Fe(CN)_6_]/K_3_[Fe(CN)_6_] (1:1) mixture (pH: 7.25) as a redox probe prepared in 10 mM PBS. The impedance was measured in a frequency range from 100 kHz to 100 mHz with a DC potential of 0.115 V versus Ag pseudo reference with a sinusoidal AC voltage of 5 mV RMS. The sampling rate was 10 points per decade. The charge transfer resistance (R_ct_) of the equivalent circuit was obtained by fitting the measured Nyquist curve using a modified Randles circuit.

First, the EIS measurement was taken on a CNT-aptamer ink-printed SPCE by placing a 50 µL droplet of the ferro-/ferri-cyanide solution on the surface of the electrode for obtaining the baseline measurement (this will be called pre-lysozyme measurement). Next, the same device was exposed to a 50 µL droplet of lysozyme of varying concentrations (0, 0.25, 0.50, 1, 2, 5, 10, and 20 µg/mL) and incubated for 15 min. The electrode was then rinsed with 50mM PBS buffer followed by rinsing in DI water to remove any unbound lysozyme protein. Afterwards, a second EIS measurement was performed to obtain the response of lysozyme binding with the aptamers (this will be called post-lysozyme measurement).

After making two rounds of EIS measurements on the same device, one for the pre-lysozyme condition and one for the post-lysozyme condition, the R_ct_ values were obtained by curve-fitting the Nyquist plot to the modified Randles circuit model. The relative change of the transduction signal ($\Delta R_{\mathrm{ct}}$) can be calculated in percentage as follows:

(1) $$\Delta R_{\mathrm{ct}}\left(\%\right)=\frac{R_{\mathrm{ct},\mathrm{post}}-R_{\mathrm{ct},\mathrm{pre}}}{R_{\mathrm{ct},\mathrm{pre}}}\times 100\%$$

where R_ct,pre_ and R_ct,post_ denote the charge transfer resistances of the pre-lysozyme and post-lysozyme measurements, respectively.

### 2.7. Chronocoulometry Experiments

To calculate the packing density of aptamers on the WE, chronocoulometry (CC) was performed by applying a pulsed voltage with a pulse width of 200 mV versus Ag pseudo reference and a pulse period of 10 s. First, the measurement was done with the sensor in 10 mM Tris-HCl buffer. Next, the sensor was incubated in 1 mM hexamine ruthenium (III) chloride (RuHex) in 10 mM Tris-HCl for 1 h. Then, the sensor was washed in DI water to remove any excess RuHex that was not bound to the DNA aptamer. Finally, the CC was performed for the RuHex incubated sensor. Following the experiment, the aptamer packing density was calculated from the CC intercepts at t=0. See Appendix C for the experimental details.

## 3. Results and Discussion

### 3.1. Patternability of the CNT-Aptamer Ink

**Figure 3:**
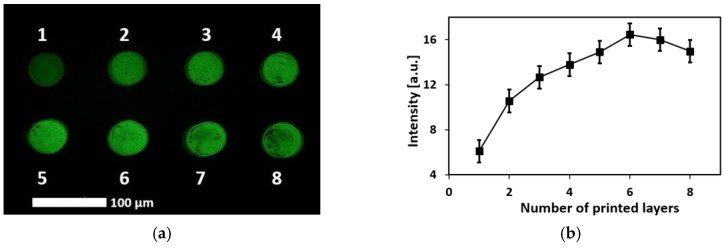

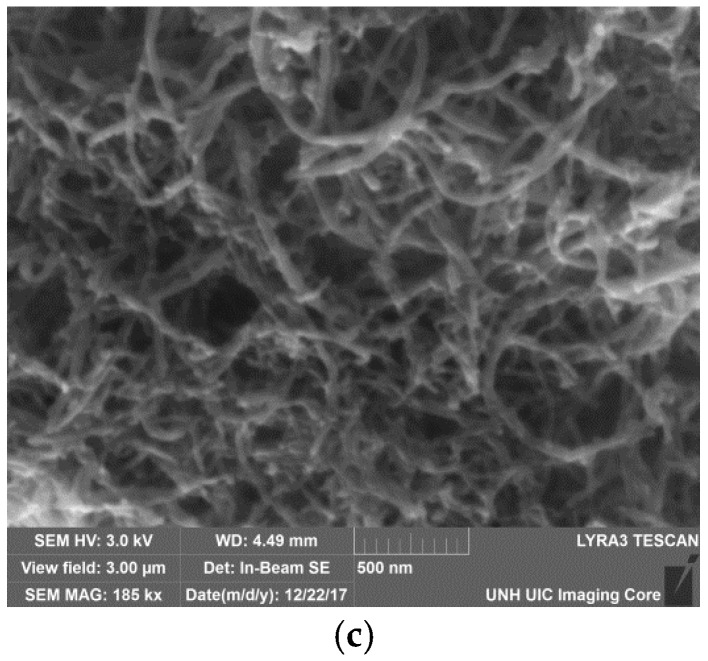
(**a**) Fluorescence image of the printed CNT-aptamer ink in a single-droplet array with different numbers of layers (as indicated by the numbers in the image); and (**b**) the intensity profile of the printed circles versus the number of layers. Each droplet has a diameter of 40 µm; (**c**) shows an SEM image of the CNT-aptamer ink used for lysozyme recognition.

The patternability of the aptamers has been characterized optically by fluorescence imaging. For the ink preparation, the aptamers were labeled with a fluorescence (6-FAM) modified at the 5′ end. Different numbers of layers (one to eight) were printed on a microporous PET transparency film as a single droplet array, as shown in Figure 3a. The droplet array was washed with DI water before imaging to remove loosely adsorbed aptamers that remained from the ink. The intensity profile of the array is presented in Figure 3b against the number of printed payers. It can be seen that, for the number of print layers from one to six, the fluorescence intensity is proportional to the number of layers. However, for seven and eight layers of printing, the intensity decreases slightly. This decrease in intensity for higher number of layers can be attributed to the possible quenching of the fluorophore due to the overcrowding of the aptamers that can lead to the cross-hybridization among neighboring aptamers, a potential result of the self-complimentary nature of the individual aptamer sequences [27]. Furthermore, the coffee ring effect [28] becomes more pronounced for higher number of layers, as can be seen in Figure 3a. In summary, we have demonstrated the ability to control the density of the immobilized aptamer by choosing the proper number of printed layers. Furthermore, a minimum patterning resolution of 40 µm was obtained with the CNT-aptamer ink. Figure 3c shows the scanning electron microscope (SEM) image of the CNT-aptamer ink, which shows well-dispersed nanostructures that allow easy access to the aptamers by lysozyme proteins.

### 3.2. Characterization of the Sensor

**Figure 4:**
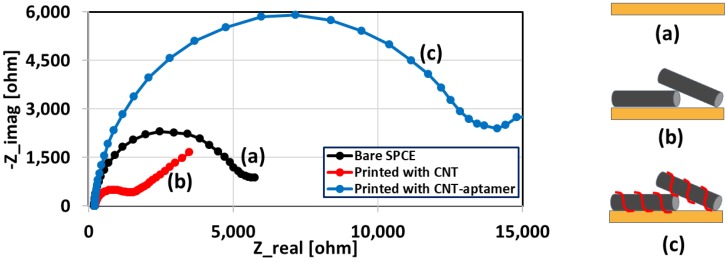
The Nyquist curves obtained with electrochemical impedance spectroscopy (EIS) measurements at different modification stages of the electrode: (**a**) bare screen-printed carbon electrode (SPCE); (**b**) printed with CNT ink; and (**c**) printed with CNT-aptamer ink. Aptamer wrapping to CNTs significantly increases the charge transfer resistance (R_ct_) due to the negative backbone of the DNA aptamers.

Figure 4 shows the Nyquist curves of the SPCE with different modifications on the working electrode: (a) bare device, (b) with CNT ink printed, and (c) with CNT-aptamer ink printed. When the electrode is printed with CNT only, the charge transfer resistance (R_ct_) decreases due to the highly conducting nature of MWCNT. However, when the electrode is printed with the CNT-aptamer ink, the R_ct_ increases significantly due to the negative charges of the single-stranded DNA oligonucleotides, as well as the electrical shielding of the CNTs by the insulating DNAs.

**Figure 5:**
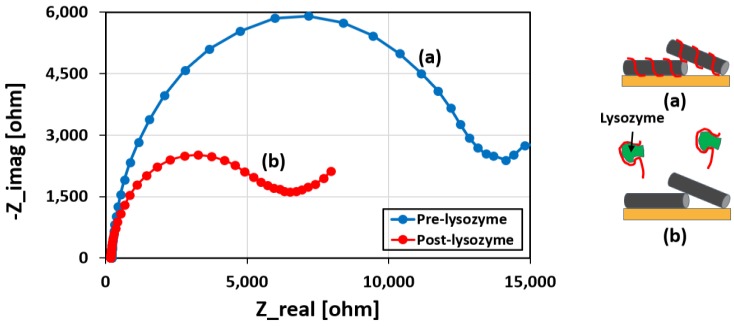
Effect of lysozyme exposure on the printed sensor. Nyquist curves for (**a**) pre- and (**b**) post-lysozyme conditions. It can be observed that lysozyme (1 µg/mL) exposure reduces the charge transfer resistance (R_ct_) because of the unwrapping of the anti-lysozyme aptamers from the CNTs to capture the lysozyme protein.

Figure 5 compares the responses of the printed sensor before and after exposure to the target protein biomarker. As can be seen from the figure, when the sensor is exposed to lysozyme, R_ct_ decreases considerably. This can be attributed to the conformational changes of the anti-lysozyme aptamers upon specific binding to the target, resulting in an unwrapping of the DNAs from the CNTs. The unwrapped aptamers are then removed from the device via rinsing the electrode, thereby decreasing the charge transfer resistance.

**Figure 6:**
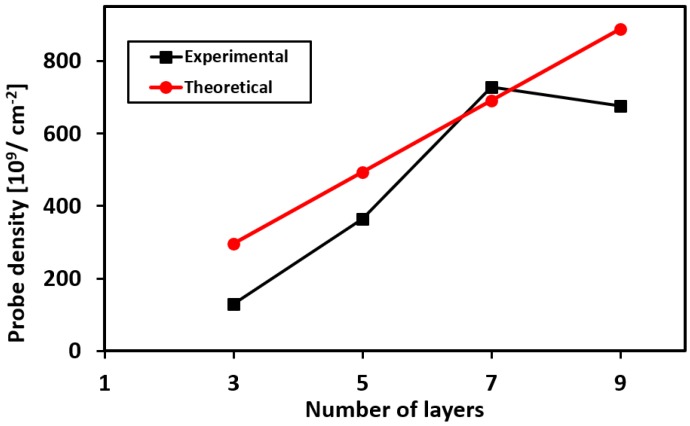
Change of charge transfer resistance (R_ct_) due to lysozyme exposure to bare SPCE (black bars) and printed SPCE (red bars) for different lysozyme concentrations.

Lysozyme binding of the aptamers was further confirmed by comparing the responses of the printed devices with those of bare SPCEs. As summarized in Figure 6, the change in R_ct_ is much larger for the printed sensor than for the bare electrode. The R_ct_ changes in the bare electrodes are likely due to the non-specific adsorption of the target biomarker on the electrode surface.

### 3.3. Packing Density of the Aptamer Probes

The amount of ink printed for five layers is approximately 1.575 µL. The theoretical number of aptamer probes printed on the working electrode can be calculated as follows:

(2) $$n=M\times V\times \left(1-\chi \right)\times 6.023\times 10^{23}$$

where *n* is the number of aptamer molecules, *M* is the molarity of the aptamers in the ink, *V* is the volume of ink printed, and *χ* accounts for the fraction of aptamers that are not attached to the CNT during the sonication-assisted dispersion of the CNT-aptamer mixture. From Equation (2), the number of aptamer molecules printed per layer on the WE (area: 0.12 cm^2^) of the electrode is 1.23 × 10^10^ molecules per layer. Hence, the aptamer density can be calculated as approximately 1.03 × 10^12^ molecules/cm^2^ per layer.

Experimentally, the probe packing density can be calculated by the integrated Cottrell equation [29]:

(3) Q=2nFAD012Coπt12+Qdl+QSE

where *n* is the number of electrons per molecule for reduction, *F* is the Faraday constant (96485.33 C/mol), *A* is the electrode area (cm^2^), *D_o_* is the diffusion constant (cm^2^/s), *C_o_* is the bulk concentration (mol/cm^3^), *t* is the time (s), *Q_dl_* is the double layer capacitive charge (C/mol) and *Q_SE_* is the surface excess charge (C/mol) from the reduction of the adsorbed redox marker. Q_SE_ is related to the density of the redox probe, $\Gamma_{0}$ (mol/cm^2^) by the following equation [16]:

(4) $$\Gamma_{0}=\frac{Q_{SE}}{nFA}$$

The value for Q_SE_ can be calculated from chronocoulometry experiments. The chronocoulometric intercept at t=0 is the sum of the double layer capacitive charge and the surface excess charge. The surface excess charge (Q_SE_) is determined from the difference in chronocoulometric intercepts (at *t* = 0) in the presence and absence of the redox probes for the identical potential steps. The density of the aptamer ($\Gamma_{DNA}$) is given by the following equation [16]:

(5) $$\Gamma_{DNA}=\Gamma_{0}\frac{z}{m}N_{A}$$

where *z* is the charge of each redox molecule, *m* is the number of nucleotides in the aptamer base sequence, and *N_A_* is Avogadro’s number.

**Figure 7:**
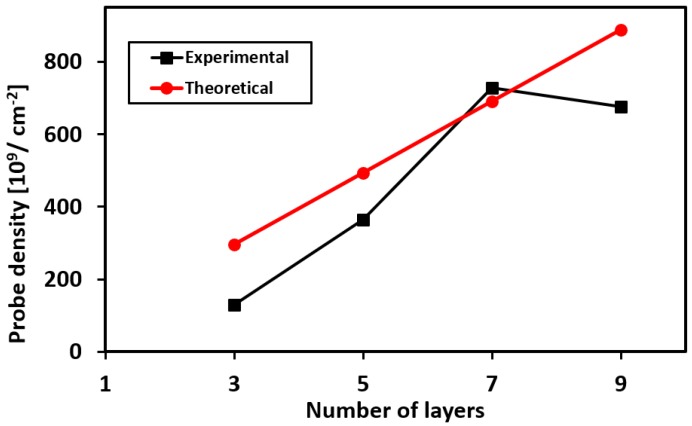
Packing density of aptamer probes as a function of number of printed layers.

Chronocoulometry was performed for different number of printed layers (see Appendix C). The calculated packing density for different number of layers is plotted in Figure 7. The graph shows that the packing density increases with increasing number of printed layers, and then saturates for further number of layers. This saturation effect can presumably be attributed to the steric and electrostatic repulsion among the negatively charged aptamers [30]. We also fitted the experimental data with the theoretically calculated values described by Equation (2) from where we can estimate the value for χ = 98.7%. Furthermore, using this value of χ, we have estimated the final concentration of the aptamers present in the printed ink as 65 nM.

The influence of the number of printed layers on the sensing performance was also characterized. It was observed that for lower number of printed layers (between one to four layers), the sensor response was relatively consistent. This is because of two opposing factors: (1) there is insufficient coverage of the ink on the electrode and therefore charge transfer would mostly occur through the regions not covered by the CNT-aptamer complex, and (2) the sensitivity is the highest for lower number of prints because the aptamers are readily available to react with the target. As the number of layers increase further, the aptamers that are buried deep in the printed ink are not able to bind with the protein and hence remain as electrical insulators, resulting in poor sensitivity. The plot in Appendix E illustrates the influence of the number of printed layers on the sensor’s sensitivity. The sensor’s response time correlates with the thickness of the printed layers, in other words, the number of prints on the electrode. In our experiments, all devices were printed five times for lysozyme detection.

### 3.4. Performance of the Aptamer Sensor

**Figure 8:**
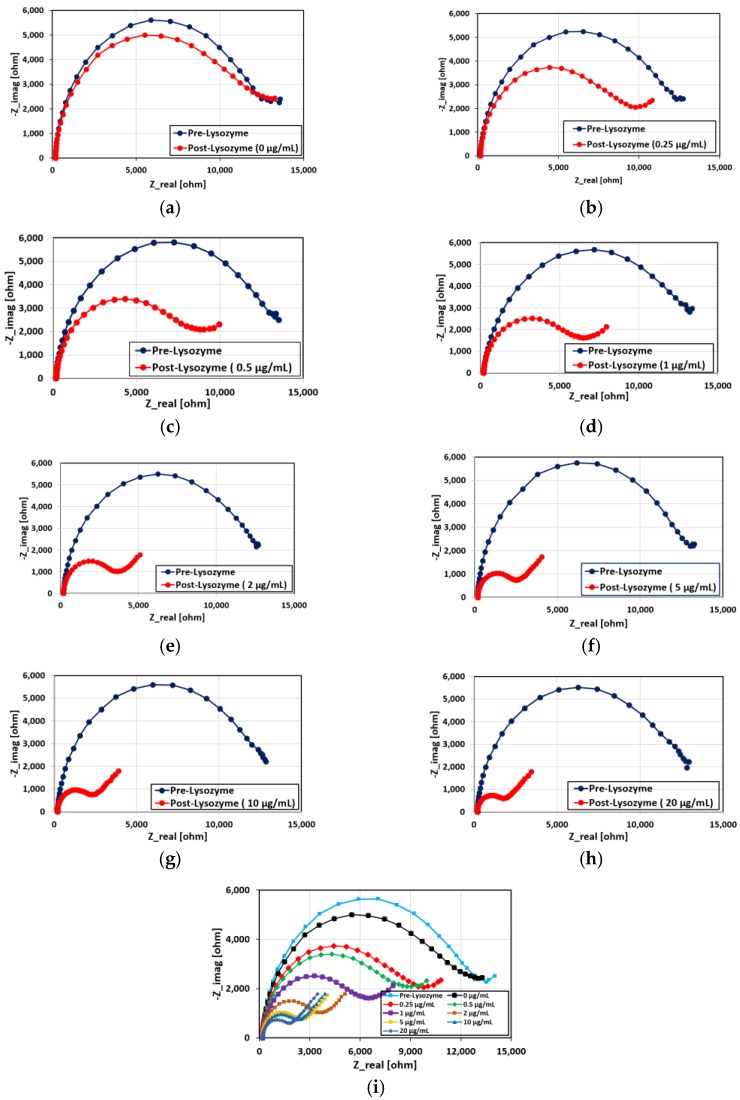
(**a**)–(**h**) Pre- and post-lysozyme Nyquist curves for different concentrations of lysozyme (0, 0.25, 0.5, 1, 2, 5, 10, 20 µg/mL, respectively) in 50 mM PBS as well as (**i**) post-lysozyme Nyquist curves for all the lysozyme concentrations.

The performance of the aptamer sensor has also been characterized by measuring the relative change in sensor response for different concentrations of lysozyme analyte. The results are presented in Figure 8 where each sensor contains five layers of printed CNT-aptamer ink.

### 3.5. Modelling of the Nyquist Curves

**Figure 9:**
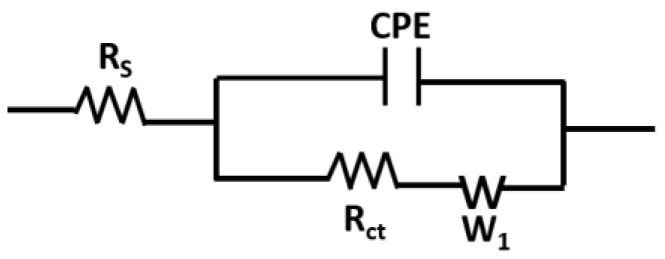
Modified Randles circuit representing the equivalent circuit model to fit the Nyquist curves of the EIS measurements.

The pre- and post-lysozyme Nyquist curves can be modelled by the modified Randles circuit shown in Figure 9, where R_S_ is the solution resistance, R_ct_ is the electron transfer resistance, CPE (constant phase element) represents the double layer capacitance at the solution–electrode interface for a rough surface [31], and W_1_ is the Warburg impedance.

The CPE accounts for the roughness of the electrode surface and, mathematically, its impedance is (Z_CPE_) described by the following equation [32].

(6) $$Z_{CPE}=\frac{1}{Q_{1}\times {\left(j\omega \right)}^{\alpha_{1}}}$$

where *j* is the imaginary unit, and α_1_ and *Q*_1_ are the characteristic parameters of the constant phase element. The introduction of the CPE instead of a simple capacitance is particularly important for the modelling of primary protein layers on the electrode surface, and the parameter α_1_ was found to vary between 0.925–0.961 for our sensor devices. For α_1_ = 1, the CPE turns into a simple capacitance. W_1_ is the circuit element corresponding to Warburg impedance resulting from the semi-infinite diffusion of ions from the bulk electrolyte to the electrode interface and is mathematically given by [33,34]:

(7) $$Z_{W1}=\frac{\sqrt{2}\;\delta_{1}}{\sqrt{j\omega }}$$

where δ_1_ is the characteristic value of the Warburg element. Table 1 summarizes the modified Randles circuit parameters of the post-lysozyme Nyquist curves of Figure 8i.

**Figure 10:**
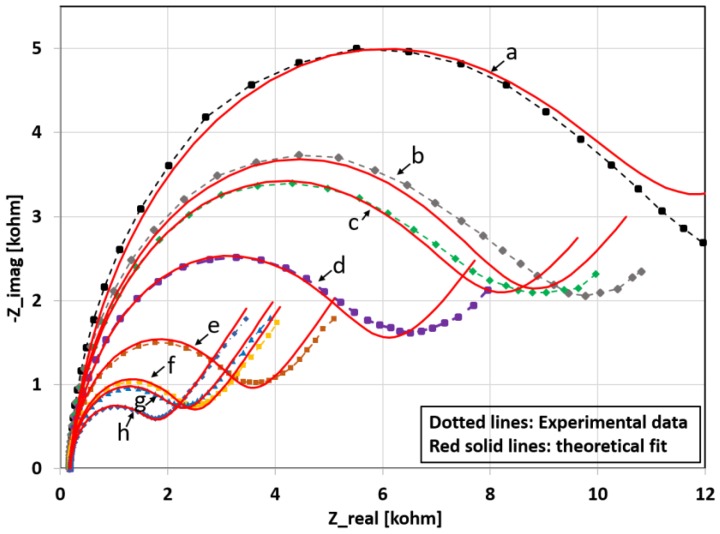
Post-lysozyme exposure Nyquist curves for different concentrations of lysozyme: (**a**) 0 µg/mL; (**b**) 0.25 µg/mL; (**c**) 0.50 µg/mL; (**d**) 1 µg/mL; (**e**) 2 µg/mL; (**f**) 5 µg/mL; (**g**) 10 µg/mL; and (**h**) 20 µg/mL. The dotted lines are the experimental data and the solid lines in red are theoretical curves based on the modified Randles circuit.

Figure 10 shows the theoretically fitted post-lysozyme Nyquist curves based on the modified Randles circuit (red solid lines). The dotted lines represent the experimental data. The graph shows good agreement between the experimentally obtained Nyquist curves and those obtained from the theoretical model.

**Figure 11:**
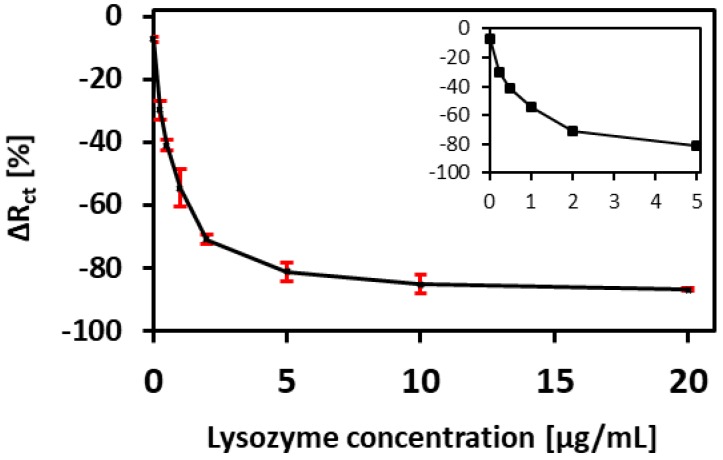
Relative change in charge transfer resistance (R_ct_) after lysozyme exposure with varying concentrations (0, 0.25, 0.50, 1, 2, 5, 10, and 20 µg/mL). Error bar shows 1 standard deviation with *n* = 3. The inset graph shows the magnified plot for low concentration range from 0 to 5 µg/mL.

The calibration curve for our aptamer-based sensor is presented in Figure 11. It shows that for low concentrations of lysozyme, the sensor exhibits high sensitivity and at higher concentrations (5 µg/mL and above) the sensor’s response reaches a saturation. Based on this calibration curve, the detection limit was calculated to be 90 ng/mL. (See Appendix D for the formula used for calculation).

### 3.6. Selectivity of the Aptamer Sensor

**Figure 12:**
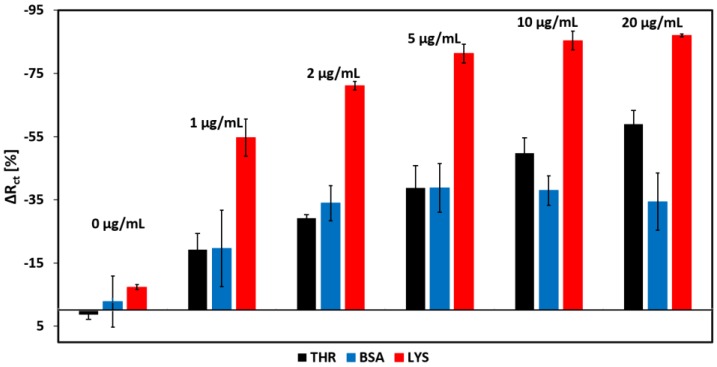
Selectivity of the aptamer biosensor to lysozyme against other proteins such as thrombin and bovine serum albumin for different concentrations.

The selectivity of our aptamer-based lysozyme sensor was investigated against two other proteins: thrombin (THR) and bovine serum albumin (BSA). It is clear from Figure 12 that our aptamer-based biosensor is highly selective toward lysozyme. The non-zero responses for THR and BSA can be attributed to the non-specific adsorption of the proteins on the sensor. Although the aptamers were designed to selectively bind with lysozyme, THR and BSA do have some level of affinity with the aptamers, resulting in a false recognition of the analyte. However, the non-specific binding efficiencies are significantly lower than that of lysozyme, hence the signal responses are markedly smaller compared to the specific target recognition by lysozyme. Better optimization of the aptamer sequence is expected to further enhance the target selectivity. For instance, a 42-mer aptamer sequence (ATC TAC GAA TTC ATC AGG GCT AAA GAG TGC AGA GTT ACT TAG) is reported to have improved binding efficiency [35], which could further reduce the response from THR and BSA.

### 3.7. Long-Term Stability (Shelf-Life) of the Aptamer-Printed Biosensor

**Figure 13:**
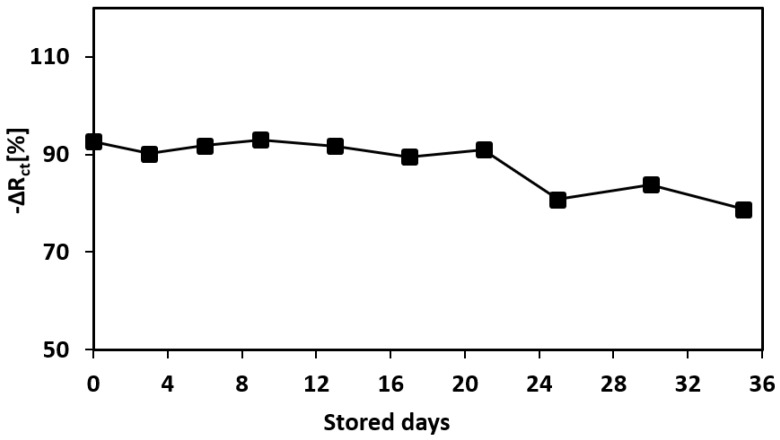
Shelf-life of the fabricated aptamer-based biosensor. The sensor response is plotted against the number of stored days at room temperature.

The shelf-life or the long-term storage stability of the developed biosensor was investigated by storing the fabricated devices for a period of up to 35 days at room temperature. After the storage period, the sensor was tested by measuring the Nyquist curves for the pre-exposure and post-exposure measurements with the lysozyme concentration of 1 μg/mL. As can be seen from Figure 13, the sensor response is reasonably consistent (with a tolerance of ±1.73%) for the first 21 days, then experiences a drop in the resistance change afterwards. Hence, it can be concluded that our proposed sensor is stable for 21 days at room temperature. However, it is expected that the shelf-life would be further extended if the devices were stored in a cooler temperature, such as at 4 °C. Moreover, because we are utilizing an inkjet-printed sensor, one advantage is that the sensor can be printed on-demand so that the storage time can be minimized.

### 3.8. Comparison to Other Aptamer-Based Lysozyme Sensors

In order to compare the sensing performance presented in this work with other recently reported lysozyme sensors, Table 2 summarizes the detection limit (LOD), linear range, immobilization method, and detection mechanism of several recently published works. The table demonstrates that the sensor presented in this paper shows comparable performances with other reported sensors. However, the main advantage and novelty of the proposed device is the convenience and the ease of immobilizing and patterning the aptamers on the electrode using the precision inkjet-printer for low-cost and disposable sensor development.

## 4. Conclusions

An inkjet-printed aptamer-based biosensor has been developed for the label-free selective detection of lysozyme biomarker. Electrochemical impedance spectroscopy was used as the interrogation method. The selectivity of the sensor was tested against BSA and thrombin and was shown to be selective towards lysozyme. The limit of detection was calculated to be 90 ng/mL. The sensor also demonstrates a reasonable shelf-life of around 21 days at room temperature. Although we have demonstrated the feasibility of inkjet printing-based sensor development for lysozyme detection, our next step in the future work is to further characterize this sensing platform with real physiological samples such as saliva or blood serum to ensure that the results can be replicated. The proposed inkjet-printed biosensor has potential applications in point-of-care diagnostics by enabling low-cost, label-free, fast detection, and on-demand printability so that patient-centered healthcare can be delivered through a disposable disease diagnostic and screening kits.

## Figures and Tables

**Table 1:** Randles circuit parameters for the post-lysozyme Nyquist curves in Figure 8i. Data extracted using Zfit program of Bio-Logic EC-Lab software.

Lys Concentration (µg/mL)	R_ct_ (Ohm∙s)	CPE (µF∙s^n−1^)	α_1_	R_s_ (Ohm∙s)	δ_1_ (Ohm∙s^1/2^)
0	10511	4.734	0.962	186	2086
0.25	7850	4.567	0.963	172	2154
0.50	7251	6.158	0.944	178	1928
1	5359	5.313	0.943	185	1833
2	3028	4.917	0.968	175	1554
5	2064	4.449	0.970	178	1501
10	1869	5.518	0.969	189	1542
20	1435	5.526	0.961	192	1491

**Table 2:** Comparison of the sensing performances of recently published lysozyme sensors.

LOD	Linear Range	Immobilization Technique	Detection Mechanism	Reference
12.09 µg/mL	0–200 µg/mL	Covalent	EIS	[2]
1.4 fg/mL	1.4 fg/mL–14 ng/mL	Thiol-Gold	SWV	[5]
7 ng/mL	14 ng/mL–1.12 µg/mL	Thiol-Gold	SPQC	[14]
0.14 fg/mL	1.4 fg/mL–6.96 pg/mL	Thiol-Gold	EIS	[16]
200 ng/mL	0–10 µg/mL	Biotin-Avidin	EIS	[19]
76.6 fg/mL	98.2 pg/mL–49.1 ng/mL	π–π stacking	DPV	[36]
0.4 pg/mL	1–50 pg/mL	Covalent	SWV	[37]
90 ng/mL	0–1.0 µg/mL	π–π stacking	EIS	This work

**Figure A1:**
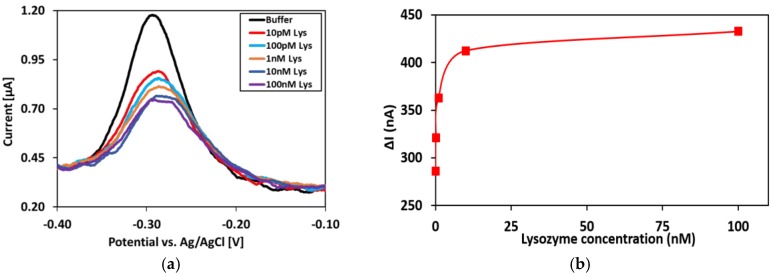
Square wave voltammograms obtained for different concentrations of lysozyme in 10 mM PBS buffer.

**Figure A2:**
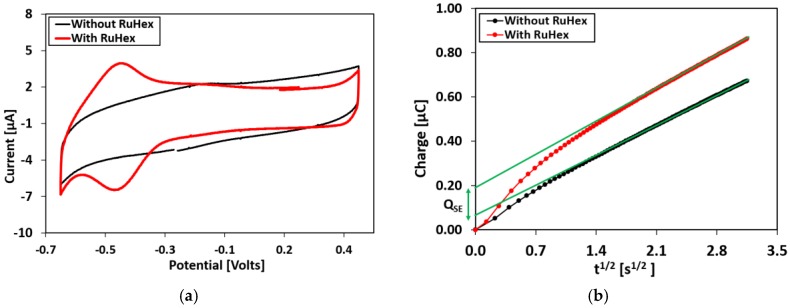
(**a**) Cyclic voltammograms (scan rate: 500 mV/s); and (**b**) CC responses curves of printed electrode in the presence (red) and absence (black) of RuHex.

**Figure A3:**
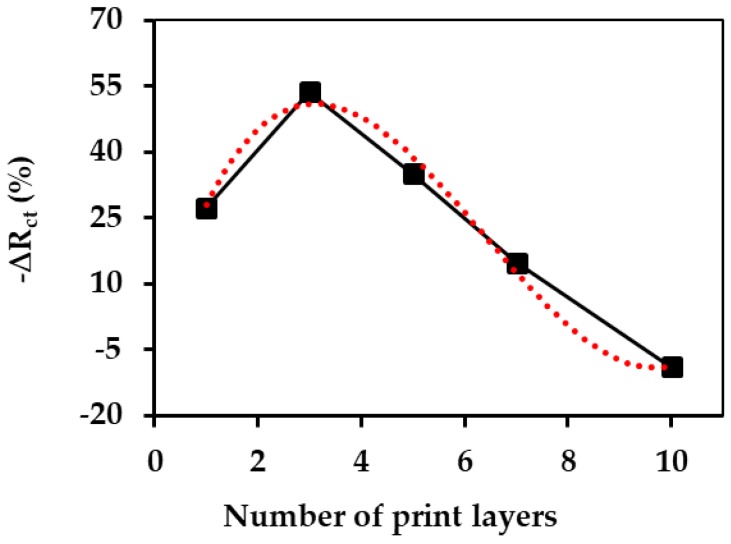
Comparison of the sensor responses as a function of the different printing layers. Each sensor was exposed to 1 µg/mL of lysozyme protein.

## Appendix A

### Calculation of Printed Volume of CNT-Aptamer Ink

Number of droplets printed on a 4 mm length = $\frac{4.0\;mm}{20\times 10^{-3\;}mm}$ = 200

Total number of droplets in the 4 mm square = (200)^2^ = 40,000

Number of droplets in the 4mm diameter circular electrode = $\frac{\pi r^{2}}{d^{2}}\times 40000=31416$, where $r=\frac{d}{2}$

Hence, the amount of ink per layer printed on the electrode = 31416×10 pL=0.31416 µL≈315 nL

## Appendix B

### Lysozyme Binding Confirmation

To confirm that lysozyme binds to the aptamer, we performed lysozyme binding experiments with MB-labelled thiolated DNA aptamers on gold rod electrode. The electrochemical DNA-based lysozyme sensor was fabricated on a 3 mm gold rod electrode (A-002421, Bio-Logic USA Science Instruments, TN, USA) using a previously described method [38]. The experiments were performed using Ag/AgCl as the reference electrode and platinum as the counter electrode. The results are presented in Figure A1.

It can be seen that, when lysozyme is exposed to the aptamer-modified gold electrode, the peak current reduces until it reaches saturation for higher concentration of lysozyme. These results suggest specific binding of lysozyme to the aptamer-based recognition element [39,40].

In the absence of lysozyme, the MB-labelled aptamer probes are relatively flexible; allowing the attached MB to collide with the electrode that enables efficient electron transfer from the MB to the electrode. This is in accordance with the relatively high voltammetric peak current for the reversible reduction of MB as characterized using square wave voltammetry (SWV). When lysozyme binds to the aptamer due to the specific affinity, the aptamer undergoes conformational change that alters the electron tunneling distance hindering the charge transfer from the MB to the electrode. As a result, the voltammetric peak current decreases.

## Appendix C

### Chronocoulometry Experiments

To perform chronoloculometry, the printed sensor was incubated in 1mM RuHex in 10 mM Tris-HCl solution for 1 h. During the incubation, RuHex ions electrostatically bind to the negative backbones of the DNA aptamers. The number of probe molecules are thus proportional to the number of bound RuHex ions to the DNA probes. After RuHex incubation, the electrode is then washed thoroughly in DI water to remove the unbound RuHex ions.

We first characterized the redox reaction of RuHex at the printed electrode using cyclic voltammetry (CV). The CV curves are presented in Figure A2(a) before and after the RuHex incubation. The two CV peaks with almost zero peak separation in the presence of RuHex indicates the electrostatically bound RuHex ions to the backbones of the surface-confined DNAs [41]. Figure A2(b) displays the CC curves at the printed electrode in the presence and absence of 1 mM RuHex. Q_SE_ is obtained from the CC intercepts at *t* = 0 and the surface density of probe DNAs can be calculated using Equations (4) and (5) in Section 3.3 where *z* = 3 and *m* = 30 in our case.

## Appendix D

### LOD Calculation

The limit of detection (LOD) can be calculated by the following equation [42]:

$$LoD=\frac{3.3\times Stabdard\;deviation\;at\;0\frac{\mu g}{mL}Lys\;concentration}{Slope\;of\;the\;calibration\;curve\;at\;0\frac{\mu g}{mL}Lys\;concentration}\;=\frac{3.3\times \;1.97}{71.89\;mL/\mu g}=90.4\;\mathrm{ng}/\mathrm{mL}$$
