# Erratum: Khan, N.I.; Maddaus, A.G.; Song, E. A Low-Cost Inkjet-Printed Aptamer-Based Electrochemical Biosensor for the Selective Detection of Lysozyme. *Biosensors* 2018, *8*, 7

**DOI:** 10.3390/bios8030058

**Published:** 2018-06-22

**Authors:** Biosensors Editorial Office

**Affiliations:** MDPI AG, St. Alban-Anlage 66, 4052 Basel, Switzerland; biosensors@mdpi.com

The *Biosensors* Editorial Office wishes to correct the following errors in this paper [1]. Figure 6 is the same as Figure 7. The correct version of Figure 6 is as follows:

In addition, there is one misspelled word, “Stabdard” in the equation in Appendix D which has been corrected to “Standard”.

The errors were made during production. We apologize for any inconvenience caused to the readers and authors by these mistakes. The manuscript will be updated and the original version will remain online on the article website.

## Figures and Tables

**Figure 6:**
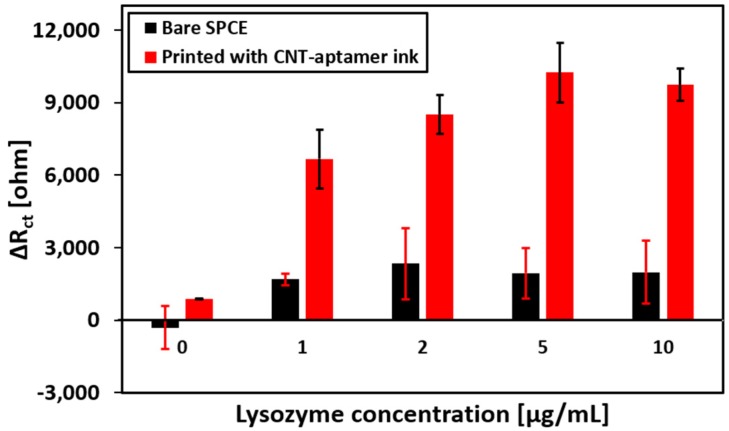
Change of charge transfer resistance (R_ct_) due to lysozyme exposure to bare SPCE (black bars) and printed SPCE (red bars) for different lysozyme concentrations.
